# Nitrogen Dioxide at Ambient Concentrations Induces Nitration and Degradation of PYR/PYL/RCAR Receptors to Stimulate Plant Growth: A Hypothetical Model

**DOI:** 10.3390/plants8070198

**Published:** 2019-06-30

**Authors:** Misa Takahashi, Hiromichi Morikawa

**Affiliations:** Department of Mathematical and Life Sciences, Hiroshima University, Higashi-Hiroshima 739-8526, Japan

**Keywords:** nitrogen dioxide, *Arabidopsis thaliana*, plant growth, cell enlargement, cell proliferation, early flowering, tyrosine nitration, PsbO

## Abstract

Exposing *Arabidopsis thaliana* (Arabidopsis) seedlings fed with soil nitrogen to 10–50 ppb nitrogen dioxide (NO_2_) for several weeks stimulated the uptake of major elements, photosynthesis, and cellular metabolisms to more than double the biomass of shoot, total leaf area and contents of N, C P, K, S, Ca and Mg per shoot relative to non-exposed control seedlings. The ^15^N/^14^N ratio analysis by mass spectrometry revealed that N derived from NO_2_ (NO_2_-N) comprised < 5% of the total plant N, showing that the contribution of NO_2_-N as N source was minor. Moreover, histological analysis showed that leaf size and biomass were increased upon NO_2_ treatment, and that these increases were attributable to leaf age-dependent enhancement of cell proliferation and enlargement. Thus, NO_2_ may act as a plant growth signal rather than an N source. Exposure of Arabidopsis leaves to 40 ppm NO_2_ induced virtually exclusive nitration of PsbO and PsbP proteins (a high concentration of NO_2_ was used). The PMF analysis identified the ninth tyrosine residue of PsbO1 (^9^Tyr) as a nitration site. ^9^Tyr of PsbO1 was exclusively nitrated after incubation of the thylakoid membranes with a buffer containing NO_2_ and NO_2_^−^ or a buffer containing NO_2_^−^ alone. Nitration was catalyzed by illumination and repressed by photosystem II (PSII) electron transport inhibitors, and decreased oxygen evolution. Thus, protein tyrosine nitration altered (downregulated) the physiological function of cellular proteins of Arabidopsis leaves. This indicates that NO_2_-induced protein tyrosine nitration may stimulate plant growth. We hypothesized that atmospheric NO_2_ at ambient concentrations may induce tyrosine nitration of PYR/PYL/RCAR receptors in Arabidopsis leaves, followed by degradation of PYR/PYL/RCAR, upregulation of target of rapamycin (TOR) regulatory complexes, and stimulation of plant growth.

## 1. Introduction

Atmospheric nitrogen dioxide (NO_2_) originates equally from natural sources, including soil microbes and lightning, and anthropogenic sources, including the combustion of fossil fuels [1,2]. Globally, atmospheric NO_2_ is a main pollutant in urban areas and a key precursor of ozone and particulate matter (PM) [3,4]. The current World Health Organization (WHO) annual guideline value for atmospheric NO_2_ is 40 µg/m^3^ (21.3 ppb) [4]. It has been reported that the average NO_2_ concentration in 141 countries is 50.6 µg/m^3^ (~27.3 ppb) [5]; this value is clearly higher than that recommended by the WHO.

Plants emit nitric oxide (NO) and NO_2_ [6,7,8,9]. Plants also absorb NO_2_ and assimilate NO_2_-derived nitrogen into amino acid nitrogen [10,11]. The compensation point concentration at which the emission of NO_2_ from plants and absorption into plants balance is reported to be 0.3–3 ppb [12]. Therefore, at 27 ppb NO_2_ (see above), plants are a sink for NO_2_. These nitrogen oxides (NO and NO_2_) are often considered as air pollutants [13]. For humans, NO_2_ at ambient concentrations is definitely toxic [14]. In contrast, the effects of atmospheric NO and NO_2_ are either toxic or non-toxic on plants contingent on their concentrations and the plant species [13,15,16,17,18]. It is noteworthy that in the nineteenth century, *von Liebig* (1827) [19] first proposed that lightning is important in the global nitrogen cycle to produce atmospheric NO and NO_2_ by the oxidation of N_2_, and that these nitrogen oxides serve as a natural fertilizer.

We discovered that atmospheric NO_2_ at ambient concentrations (10–50 ppb) acts as a stimulant signal for plant growth. We also sought understanding of why an air-pollutant such as NO_2_ can act as a stimulant factor for plant growth. Recently, based on previous research, we devised a hypothesis to answer this fundamental question, which is described below.

## 2. Nitrogen Dioxide at Ambient Concentrations of 10–50 ppb Acts as a Positive Plant Growth Signal in *Arabidopsis Thaliana*

Initially, we investigated the potential of plant material to mitigate atmospheric NO_2_ [20,21,22]. We found a higher than 600-fold difference in the assimilation ability of NO_2_ among 217 plant taxa [20]. We investigated hypothetical air-pollutant-philic plants [23,24] that utilize NO_2_ as the sole nitrogen source. During our research, we discovered that atmospheric NO_2_ at concentrations as low as 10−50 ppb positively regulates plant growth [25,26,27,28,29,30,31,32].

*Arabidopsis thaliana* (Arabidopsis) was grown in air without NO_2_ for the first week after sowing, and then for 1–4 weeks in air with (abbreviated as +NO_2_-treated plants) or without (–NO_2_ control plants) NO_2_ [26]. Seedlings were watered semiweekly with half-strength inorganic salts of Murashige and Skoog (M&S) medium [33] containing 19.7 mM nitrate and 10.3 mM ammonium. Plant age is expressed in weeks after sowing and corresponds to the time of harvest. The plant type utilized was accession C24 or Columbia. Their responses in terms of biomass increase and flowering time to NO_2_ were very similar qualitatively, but differed quantitatively (see below) [28].

**Figure 1:**
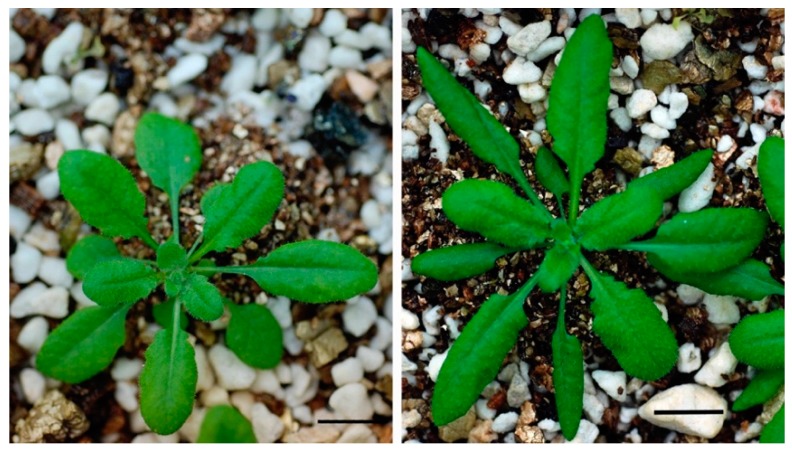
Typical 4-week-old plants of *Arabidopsis thaliana* accession Columbia grown in the presence (right) or absence (left) of 50 ppb nitrogen dioxide (NO_2_). Bar = 1 cm.

NO_2_ concentration effect on the yield of shoot biomass in 4-week-old plants was first determined. Shoot biomass of +NO_2_-treated C24 plants under 10 ± 0.2 and 50 ± 0.3 ppb NO_2_ was 3.2-fold [29] and 2.5-fold greater relative to the –NO_2_ control plants. Treatments of 100 ± 20 and 200 ± 50 ppb NO_2_ produced no stimulation of growth, or somewhat repressed the growth of plants. In this study, 50 ± 0.3 ppb NO_2_ treatments were used. Images of typical 4-week-old + NO_2_ and –NO_2_ control plants (Columbia) are shown in Figure 1.

Increase in shoot biomass by NO_2_ treatment was accompanied by increase in uptake of seven major elements, such as carbon(C), N, phosphorus (P), potttasium (K), calcium (Ca), magnesium (Mg) and sulfur (S) into shoots. The contents of these elements per shoot dry weight (DW) were virtually the same for +NO_2_-treated plants and –NO_2_ control plants, and the contents of these elements per shoot were two times greater in +NO_2_-treated plants than in –NO_2_ control plants (Table S1) [26]. These findings agreed with our previous work on *Nicotiana plumbaginifolia* [24] and other vegetable plant species [27].

*Arabidopsis thaliana* accession Columbia also increased shoot biomass in response to NO_2_ treatment as in the case of accession C24 [26]. This is consistent with the report of Xu et al. (2010) [34]. Columbia grew faster than C24, and 4-week-old Columbia appeared to be close to the end of the vegetative growth. Shoot biomasses of +NO_2_-treated plants and –NO_2_ control plants of 4-week-old Columbia were 24.2 ± 5.5 and 14.3 ± 2.5 mg (mean ± SD, n = 5), respectively [26]. This difference in shoot biomass (1.7-fold) was smaller than that in C24 (2.5-fold, see above).

NO_2_ treatment significantly shortened flowering time in both the C24 and Columbia accessions. In accordance with Kotchoni et al. [34], the number of days after sowing when the flower bolts became 1 cm long was a measure of flowering time.

The accession C24 exhibited a median flowering time of 41 and 42 d in +NO_2_-treated and –NO_2_ control plants, respectively. This difference was statistically significant by Student’s *t*-test (*P* < 0.05) [25]. In the case of the accession Columbia, flowering time was remarkably shortened by NO_2_ treatment. The median flowering time of this accession was 34 and 40 d in +NO_2_-treated plants and –NO_2_ control plants, respectively [31]. This was statistically significant (*P* < 0.001) by Student’s *t*-test [25]. A similar flowering acceleration was observed in other plants. NO_2_ treatment shortened the flowering time by 3.2 days and increased fruit yield by 1.4 times in tomato [31]. In addition, NO_2_ has accelerated the flowering of mulkhiya plants [35].

NO at the same concentration as NO_2_ increased shoot biomass in Arabidopsis C24 [25,26] and Columbia [36]. This agrees with those reports that sodium nitroprusside, a NO donor, accelerates vegetative growth of Arabidopsis [37], and that NO gas stimulates the expansion of leaf discs of pea [38] and the vegetative growth of spinach [39].

As NO_2_ stimulate shoot biomass production, we expected a similar NO_2_ effect on the root biomass production. Among 6 plant species we studied so far, 2 showed NO_2_-stimulated root biomass production, but the remaining 4 showed no NO_2_-stimulated root biomass production: Sunflower [27] and Arabidopsis C24 [25] plants that were exposed with NO2 exhibited 0.4 ± 0.04 and 4.8 ± 0.08 (g/plant) root biomass (mean of 3 or 10 plants, respectively, ± SD) which were significantly higher (*P* < 0.05 or 0.001 by Student’s t test) than corresponding value of non-exposed plants (0.2 ± 0.08 and 2.5 ± 0.6). However, NO_2_ showed no statistically significant increases in the root biomass in lettuce, cucumber, pumpkin [27] and *Nicotiana plumbaginifolia* [24]. The causes and mechanisms for this result are completely unknown, and will be an important and intriguing subject for future studies. Interestingly, NO_2_ did stimulate the seed production of mulkhiya plants [35] although whether NO_2_ exhibits similar effects on other plant species such as Arabidopsis is not known yet. Similarly, how NO_2_ stimulates other aspects of whole life cycle of plants also is an important and intriguing subject of the future studies.

To investigate the physiological role of N derived from NO_2_ (NO_2_–N), Arabidopsis seedlings were fed with ^15^N-labeled gaseous NO_2_ (50 ppb) and unlabeled nitrate (19.7 mM) and ammonium (10.3 mM), and mass spectrometric N analysis [40] including the ^15^N/^14^N ratio analysis on the aboveground parts of plants was performed. The ^15^N/^14^N ratio is a measure of the content of NO_2_–N as a relative amount of the total plant N. We found that NO_2_–N occupied < 5% (4.05 ± 0.75%; mean ± SD, n = 3) of the total N in the +NO_2_-treated Arabidopsis C24 shoots. Therefore, NO_2_–N plays only a minor role as an N source, but instead plays an important role as a plant growth signal. Similar results indicating that NO_2_ plays an important role as a plant growth signal were obtained in *Nicotiana plumbaginifolia* [24] and other vegetable plant species [27].

The increased total leaf area following NO_2_ treatment (Table S1) indicated that NO_2_ treatment increases the sizes of individual leaves. Therefore, the sizes of individual rosette leaves in positions 1–25 on 5-week-old +NO-treated plants and –NO_2_ control plants, which had 28 and 25 rosette leaves, respectively, were measured. Leaves 1–11 and leaves 12–25 were in almost maturity stages and developing stages, respectively. Each of rosette leaves was separated by an angle of approximately 137° [41]. The oldest leaf located at the bottom (root side) was numbered as leaf 1, and progressively leaves were numbered as leaf 2, 3, 4 etc. to the youngest one located at the tip of the stem (close to apical meristem) as leaf 25 or 28. Leaves 1–11 and leaves 12–25 or 28 were in almost maturity stages and developing stages, respectively.

**Figure 2:**
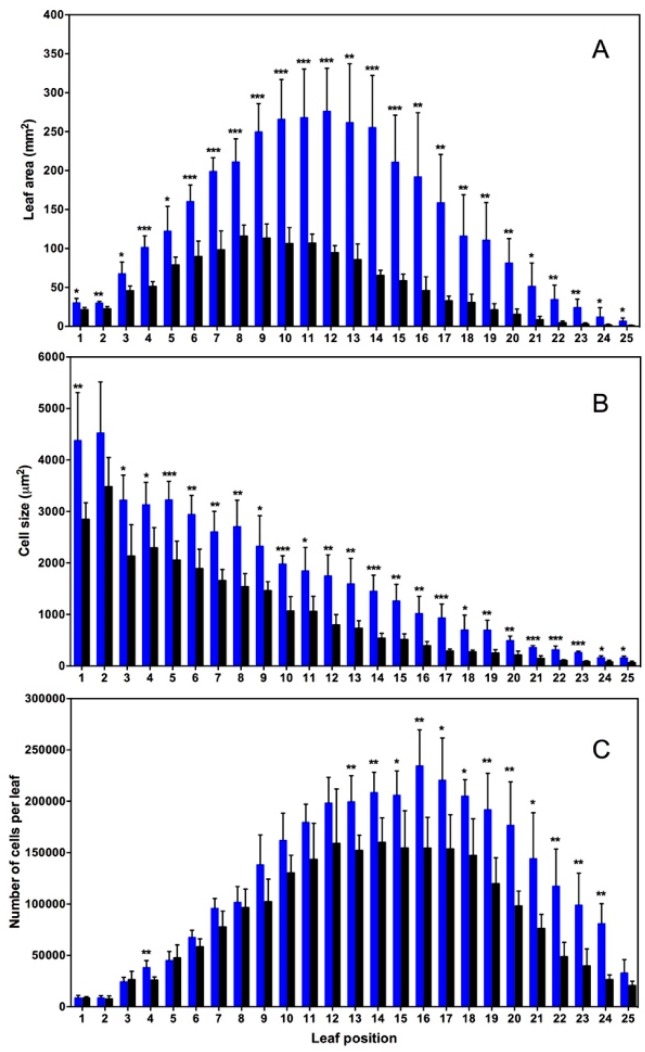
Area of leaves (**A**), size of cells (**B**), and number of cells (**C**) in 5-week-old *Arabidopsis thaliana* C24 plants as a function of leaf position. Plants were grown in the presence (+NO_2_-treated plants, blue columns) or absence (–NO_2_ control plants, black columns) of NO_2_. Values are expressed as means ± SD; n = 5. Statistical significance assessed by Student’s *t*-test (* *P* < 0.05; ** *P* < 0.01; *** *P* < 0.001).

Microscopic study was performed according to Tsuge et al. (1996) [42]. The leaves of Arabidopsis C24 plants were fixed with a FAA solution (formaldehyde-acetic acid-ethanol), and microscopic observations were carried using a stereo microscope and a differential interference microscope. Microphotographs were taken to measure leaf area, cell number, and cell size (Figure 2). Leaves 1 (the oldest) to 25 (the youngest) in +NO_2_-treated plants had 1.3–8.4 times greater leaf areas compared with –NO_2_ control plants in the corresponding leaf positions (Figure 2A). The observed differences were significant statistically at all positions according to the Student’s *t*-test (Figure 2A) [25].

It is known that determinants of organ size are cell number and cell size [43,44,45]. Therefore, we investigated whether the increases in leaf areas following NO_2_ treatment were ascribable to increases in cell numbers or cell sizes, or both. In both the +NO_2_-treated and–NO_2_ control plants of Arabidopsis, palisade cells in the adaxial sub-epidermal layer were neatly aligned in the paradermal plane throughout leaf development [42], as reported previously [25]. Thus, the sizes and numbers of palisade cells in leaves of positions 1 to 25 in 5-week-old +NO_2_-treated plants and –NO_2_ control plants of Arabidopsis C24 were determined. (Figure 2B,C).

Leaf area as a function of leaf position was more or less asymmetric in both –NO_2_ control (black columns) and +NO_2_-treated plants (blue columns) (Figure 2A); –NO_2_ control plants exhibited a positively-skewed bell-shaped pattern, while +NO_2_-treated plants exhibited less skewed and less asymmetric pattern [25]. The ratio of leaf area of +NO_2_-treated plants to that of –NO_2_ control plants at the corresponding leaf positions (designated RLA) was calculated [25]. The RLA varied from 1.3–2.5 in leaves 1–11, while varied from 2.9–8.4 in leaves 12–25. This difference in RLA between leaves 1–11 and 12–25 was significant (*P* < 0.01), as assessed by Mann-Whitney U test [25].

The size of the cells decreased as a linear function of leaf position in plants with or without NO_2_ treatment (Figure 2B). This suggests that increase in cell size is a linear function of leaf age. Presence or absence of NO_2_ did not affect this result. Student’s t test showed that these results in the cell size at all positions, except leaf 2, were statistically significant (Figure 2B) [25].

The ratio of the cell size of +NO_2_-treated plants to that of the –NO_2_ control plants at the corresponding leaf position (designated RCS) was calculated. The RCS of leaves 12–25 (2.0–3.2) was larger than that of leaves 1–11 (1.3–1.9). This difference in RCS between leaves 12–25 and 1–11 was significant statistically (*P* < 0.01) by Mann-Whitney U test. This finding is in line with our previous observation that NO_2_ exerts a greater effect on leaf expansion in younger leaves than in older leaves [24].

Cell number as a function of leaf position exhibited a normal distribution for both the +NO_2_-treated plants and–NO_2_ control plants (Figure 2C). Plants with or without NO_2_ treatment did not show significant differences in cell numbers in almost all maturing leaves (positions 1–11, except leaf 4). This suggest that the NO_2_ treatment did not affect cell numbers in the leaves in maturity. Almost all younger leaves (positions 13–24 of leaves 12–25) showed statistically significant differences in cell number, suggesting that NO_2_ did increase cell numbers in developing leaves.

The ratio of the cell number of the +NO_2_-treated plants to that of the –NO_2_ control plants at the corresponding leaf position (designated RCN) was calculated. Leaves 12–25 had larger RCN (1.2–3.1) than that of leaves 1–11 (0.9–1.5). This difference in RCN between leaves 12–25 and 1–11 was found to be significant (*P* < 0.01) by the Mann-Whitney U test [25]. This observation indicated that the effect of NO_2_ on cell proliferation changed depending on developmental stage of the leaves, and was greater in younger leaves than in older leaves.

To investigate the correlations between ratio in leaf area and the ratio in cell size (or cell number), log(RLA), log(RCS) and log(RCN) values were calculated and analyzed using Pearson’s correlation analysis and Bonferroni’s correction (Table S2). The correlation between leaf area and cell size was found to be high and significant in leaves 1–25 (R = 0.9, *P* < 0.001). Interestingly enough, the correlation between leaf areas and cell size was found to be stronger in older leaves than in younger leaves; R = 0.7, *P* < 0.05 for 1–11 leaves, while R = 0.3, *P* > 0.5 for 12–25 leaves (Table S2). This means that the correlation between NO_2_-induced leaf expansion and cell size expansion was higher in older leaves than in younger leaves. Leaf area and cell number in leaves 1–25 were found to have a significantly high correlation (R = 0.9, *P* < 0.001). The same was found to be true when developing (12–25) (R = 0.9, *P* < 0.001) and maturing (1–11) (R = 0.7, *P* < 0.05) leaves were separately analyzed (Table S2) [25].

Thus, NO_2_-induced leaf expansion correlated well with cell proliferation in both younger and older leaves. It is concluded that NO_2_-mediated leaf expansion can largely be ascribed to cell proliferation in younger leaves, while the NO_2_ effect can be ascribed to both cell proliferation and enlargement in older leaves [25].

## 3. NO_2_ Selectively Nitrates Specific Cellular Proteins in Arabidopsis Leaves

Nitration of protein tyrosine is the addition of a nitro group on the carbon-3 of tyrosine residues of proteins to produce 3-nitrotyrosine (3-NT), which accompanies a drastic decrease (from 10.0 to 7.2) in the pKa of the tyrosine hydroxy group. Protein tyrosine nitration is an important post-translational modification in cell physiology, including cellular signaling [46,47]. According to a free radical mechanism [46,47,48], prior to their nitration, tyrosine residues are oxidized to tyrosyl radicals by an oxidation mechanism. Tyrosyl radicals undergo rapid radical-radical combination with NO_2_ radicals that exist in the close vicinity of the tyrosyl radicals to produce 3-NT. Nonetheless, biological protein nitration is not a simple chemical process, but is instead a characteristic selective process in which only a restricted number of proteins are nitrated [46,47,48]. 

Selectivity of protein nitration is central for protein nitration to play a vital role in signal transduction that reflects the cellular redox state [46,47,48,49,50]. Selectivity of protein tyrosine nitration has been investigated mainly in mammals [45,46,47,50,51]. Although a number (12–127 kinds) of plant proteins are reported to be nitratable [47,51,52,53], experimental substantiation on this issue in plant protein nitration is rather scarce. NO_2_ is a potent nitrating agent that nitrates tyrosine residues on proteins to yield NT [54,55] (see Section 4). Furthermore, NO_2_ is a hydrophobic molecule (less hydrophobic than NO but more so than carbon dioxide), and thus is almost freely permeable to cell membranes [56]. In addition to its signaling role in plant growth, NO_2_ is an in vivo intermediate involved in biological protein tyrosine nitration in animals [51] and plants [49]. Therefore, we used NO_2_ as a nitrating agent; for the sake of facilitating nitrated protein and nitration site identification, plants were exposed to high (4–40 ppm) concentrations of NO_2_ [57]. 

Arabidopsis (accession C24) plants were exposed to air containing or not containing 40 ppm NO_2_ for 8 h under illumination. Proteins were extracted from whole leaves (abbreviated as whole leaf protein). Alternatively, chloroplasts were isolated and fractionated into soluble (stromal and lumenal) and insoluble (thylakoid membrane) fractions, and proteins were extracted from each fraction (abbreviated as chloroplast protein) [57]. Proteins were analyzed using two-dimensional polyacrylamide gel electrophoresis (2D PAGE), followed by staining with SYPRO Ruby stain and Western blotting using a 3-NT-specific antibody.

**Figure 3:**
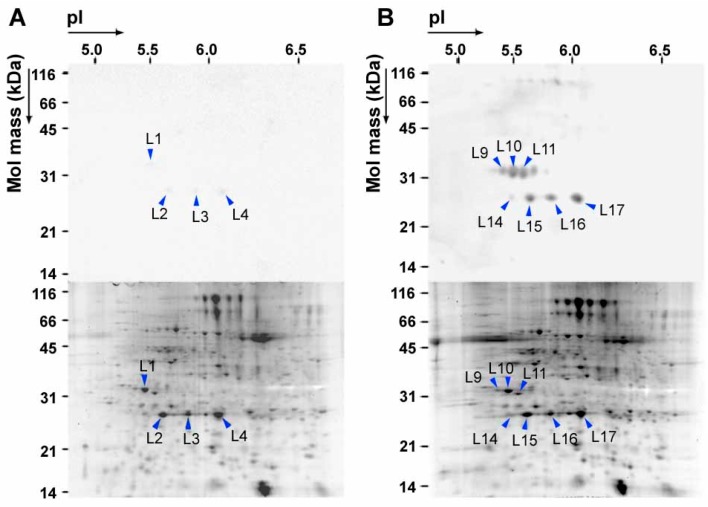
2D PAGE gel patterns of Arabidopsis whole leaf proteins extracted from +NO_2_-treated (right) and –NO_2_ control (left) plants. Western blots detected using 3-NT-specific antibody (upper panels) and gels stained with SYPRO Ruby (lower panels). Each gel was loaded with 100 µg protein.

**Figure 4:**
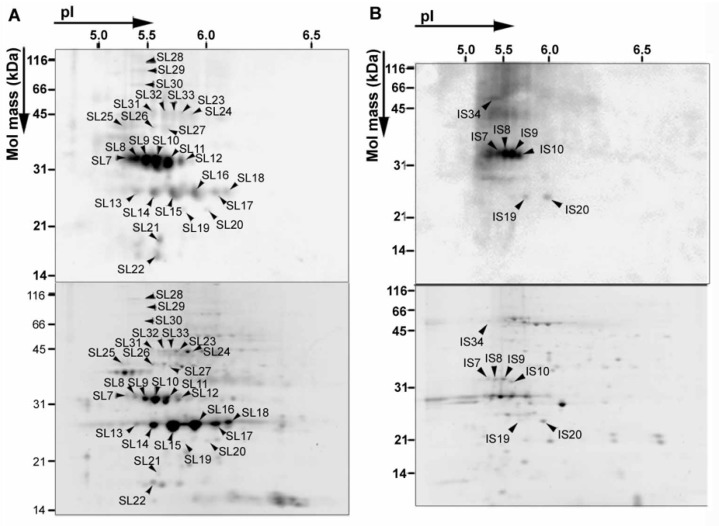
2D PAGE gel patterns of chloroplast proteins extracted from +NO_2_-treated plants. The stromal and lumenal protein fraction (**A**), and the thylakoid membranous protein fraction (**B**) are shown. Spots on gel (**A**) and (**B**) were numbered as SLn and ISn, respectively. Upper and lower panels of (**A**) and (**B**) correspond to Western blots detected using 3-NT-specific antibody and gels stained with SYPRO Ruby, respectively. Each lane of stromal and lumenal protein fraction and thylakoid membranous protein fraction was loaded with 40 and 20 μg protein, respectively.

The 2D PAGE images of whole leaf proteins and chloroplast proteins are shown in Figure 3 and Figure 4, respectively. The relative intensities of spots on Western blots (abbreviated as RISI), and those of the spots on SYPRO Ruby gels (abbreviated as RISS) were determined. Nitrated proteins identified in chloroplast protein fractions and their electrophoretic and proteomic characteristics are summarized in Table S3. Proteins that showed a high RISI and/or a high RISI/RISS were concluded to be selectively nitrated [57]. Seven 3-NT-positive spots were detected on a Western blot of whole leaf proteins from exposed leaves (Figure 3), all of which were identified as PsbO1, PsbO2 or PsbP1 by peptide mass fingerprinting (PMF) [57].

PsbO and PsbP are external proteins localized on the stromal side of the thylakoid membrane in PSII. PsbO and PsbP stabilize the oxygen-evolving complex (OEC) of PSII together with other external proteins, including PsbQ and PsbR [58,59,60]. No nitration of PsbQ or PsbR was detected. Thus, nitration was specific to PsbO and PsbP, while their RISI/RISS ratio was low (≤ 1.5) (Table S3). Non-exposed control plants exhibited very faint 3-NT-positive spots.

The number of 3-NT-positive spots was markedly increased in purified and fractionated chloroplast proteins (Figure 4, Table S3) [57]. Distinct 3-NT-positive protein spots were lined at 32 kDa (SL7–12), and distinct but clearly visible spots were lined at 27 kDa (SL13–18) on the Western blot of the soluble (stromal and lumenal) chloroplast protein fraction from +NO_2_-treated plants (Figure 4A, upper panel). Lined spots of less in number at 32 kDa (IS7–10) were detected on the Western blot of the thylakoid membrane protein fraction from +NO_2_-treated plants (Figure 4B, upper panel). PsbO and PsbP accounted for > 80% of the total RISI values [Table S3], and high RISI/RISS ratios (2.5–6.6) were exhibited by four non-PSII proteins such as peroxiredoxin II E (PRXII E) (spot SL21), thylakoid lumenal protein (SL22), RuBisCO activase (RCA, SL31), and the delta subunit of chloroplast ATP synthase (SL19) [Table S3]. Thus, PsbO, PsbP and these four non-PSII proteins are concluded to be selectively nitrated.

Despite that use of purified/fractionated chloroplast proteins markedly increased the number of 3-NT positive spots on Western blots (Figure 4), no 3-NT-positive spots attributable to other extrinsic (such as PsbQ and PsbR) or intrinsic (such as D1 and D2) proteins of PSII were detected, and nor were 3-NT-positive spots attributable to RuBisCO subunits (Figure 4, Table S3). Thus, NO_2_ selectively nitrates two PSII and four non-PSII proteins in Arabidopsis. PMF analysis using MALDI-TOFMS provided evidence that the ninth tyrosine residue (^9^Tyr) of PsbO1 is a nitration site [57].

## 4. PsbO1 May Function as an Electron Element Like Yz in PSII Electron Transport Chain

To investigate the physiological significance of protein nitration, thylakoid membranes were isolated from Arabidopsis leaves and incubated in a buffer solution bubbled with NO_2_ gas or a buffer solution of potassium nitrite (KNO_2_). The former buffer contains NO_2_ and nitrite (NO_2_^−^), while the latter contains NO_2_- alone [61]. NO_2_ dissociates in water as shown in reaction 1 [62], as described previously [61]. Concentrations of NO_2_ in the buffer were quantified by numerical solution of kinetic Equations (1)–(3). Nitrite (NO_2_^–^) concentrations in the buffer were quantified by capillary electrophoresis [63].

Reaction (1) $${\mathrm{NO}}_{2}\begin{array}{c} \overset{k_{1}}{\to }\\ \underset{k_{2}}{\leftarrow } \end{array}\;N_{2}O_{4}\overset{k_{3}}{\to }{\mathrm{NO}}^{2-}+{\mathrm{NO}}^{3-}$$

(1) $$\frac{d\left[{\mathrm{NO}}_{2}\right]}{dt}=-2k{\left[{\mathrm{NO}}_{2}\right]}^{2}+2k_{2}\left[N_{2}O_{4}\right]$$

(2) $$\frac{d\left[N_{2}O_{4}\right]}{dt}=k_{1}{\left[{\mathrm{NO}}_{2}\right]}^{2}-k_{2}\left[N_{2}O_{4}\right]-k_{3}\left[N_{2}O_{4}\right]$$

(3) $$\frac{d\left[{\mathrm{NO}}_{2}{}^{-}\right]}{dt}=k_{3}\left[N_{2}O_{4}\right]$$

where *k*_1_, *k*_2_ and *k*_3_ are rate constants 4.5 × 10^8^ mol^−1^ s^−1^, 6.4 × 10^3^ s^−1^, and 10^3^ s^−1^, respectively [62].

**Figure 5:**
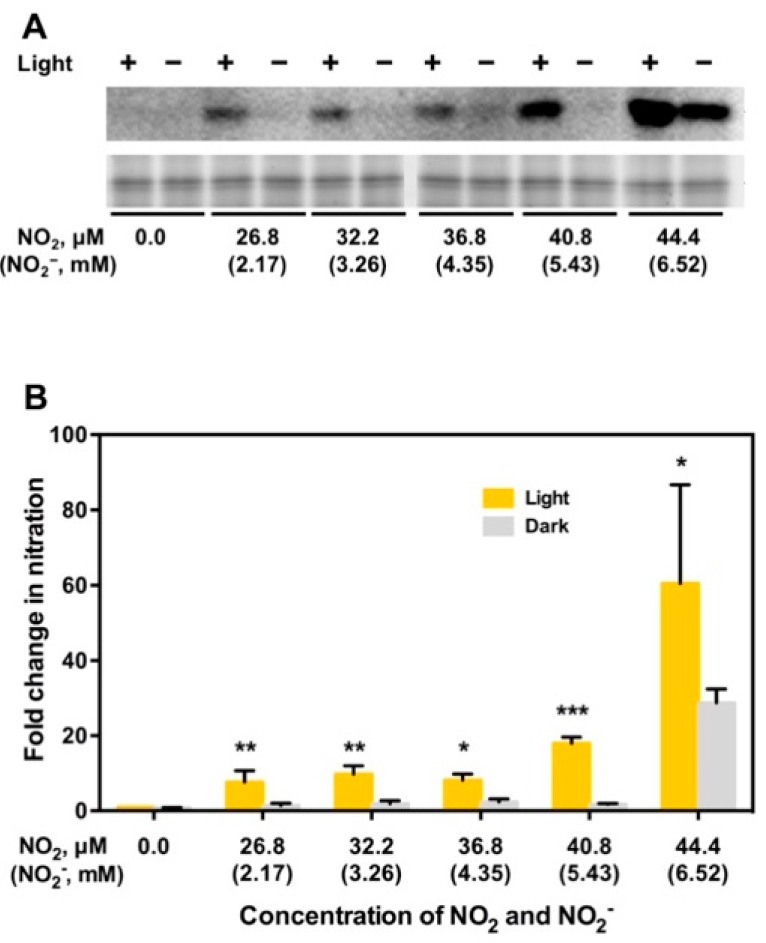
Demonstration that light is essential to induce nitration of PsbO1. (A) Arabidopsis thylakoid membranes were incubated in a buffer containing NO_2_ and NO_2_^−^ with or without illumination. Upper and lower panel show 3-NT-positive band and SYPRO-Ruby-stained band of PSBO1, respectively. (B) Fold-change in the PsbO1 band intensity (FCPSBO1) as a function of NO_2_ and NO_2_^−^ concentrations in a buffer solution bubbled with NO_2_ gas. FCPSBO1 = (PsbO1 band intensity following incubation in a buffer bubbled with NO_2_ gas)/(PsbO1 band intensity following incubation in buffer without NO_2_ or NO_2_^−^). Data represent means of 3 independent experiments ± SD. *, *P* < 0.05; ***, *P* < 0.001. Student’s *t*-test was done using GraphPad Prism 6.0 (GraphPad Software, La Jolla, CA, USA).

A distinct 3-NT-positive band of 32.5 kDa was detected on a Western blot of proteins extracted from thylakoid membranes that were incubated in a buffer containing NO_2_ and NO_2_^−^ under illumination (Figure 5A). This band was assigned to PsbO1 by liquid chromatography/mass spectrometry (LC/MS), followed by a Mascot search analysis [64]. On the other hand, no such band was detected following incubation thylakoid membranes in the same buffer in the dark at all concentrations of NO_2_ and NO_2_^−^ (Figure 5). Thus, illumination is essential in NO_2_/NO_2_^−^-mediated protein nitration. The intensities of the PsbO1 band on the Western blots were quantified using PDQuest software (ver. 7.0; Bio-Rad, Hercules, CA, USA) [64]. The intensity of the 3-NT-positive PsbO1 band after incubation in a buffer containing NO_2_ and NO_2_^−^ was divided by the intensity of the 3-NT-positive PsbO1 band before incubation in the buffer. This value was designated fold-change in the PsbO1 band intensity, and plotted against the concentrations of NO_2_ and NO_2_^–^ (Figure 5B). Incubation in the dark resulted null intensity of PsbO1 band at all concentrations of NO_2_ and NO_2_–except 44.4 μM NO_2_ and 6.52 mM NO_2_^−^ (Figure 5). This confirms that illumination is essential in NO_2_/NO_2_^–^-mediated protein nitration of PsbO1 in Arabidopsis thylakoid membranes.

Redox-active tyrosines play a key role in the photosynthetic electron in PSII. Yz (161Tyr of the D1 protein) in PSII is the most well-studied redox-active tyrosine residue in plants. Under illumination, it donates an electron to the PSII electron transport chain and itself is oxidized to tyrosyl radical [65,66]. It is reduced back to tyrosine by an electron derived from oxidation of water at the OEC. Thus, Y_z_ functions as an electron relay element between P680 and OEC Mn_4_ cluster (Mn_4_Ca) through photosynthetic electron transfer [67]. Another tyrosine that has a similar function, Y_D_ (161Tyr of the D2 protein), is also known [65,66]. 

In light of our finding of the illumination-triggered nitration of ^9^Tyr of PsbO, it is conceivable that this tyrosine residue of PsbO1 is also redox-active, and that the photosynthetic electron transport chain can oxidize, upon illumination, this tyrosine residue to tyrosyl radical that is highly sensitive to nitration. The formed tyrosyl radical may rapidly react with NO_2_ to yield 3-NT. Therefore, we hypothesized a nitration mechanism that prior to nitration PSII photosynthetic electron transport, in response to illumination, oxidizes the nitratable tyrosine residue of PsbO1 to tyrosyl radical to react with NO_2_ to yield 3-NT [64].

**Figure 6:**
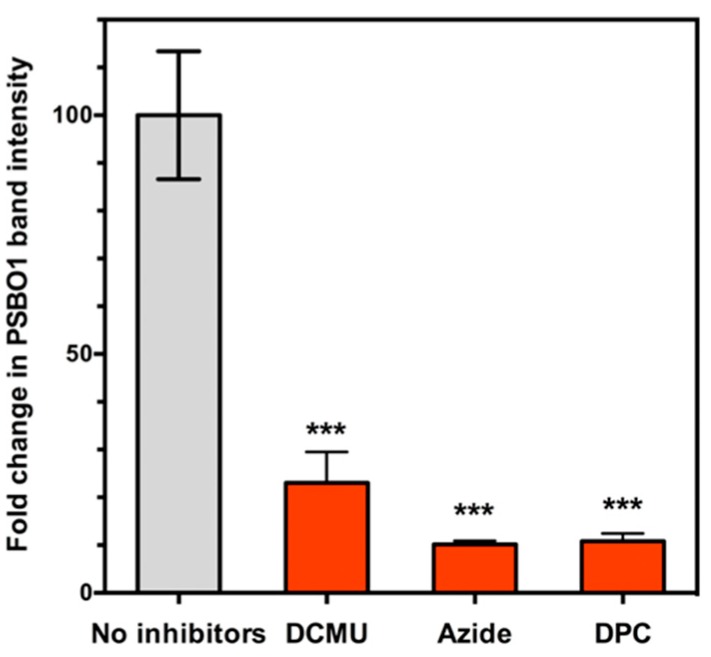
Demonstration that PSII electron transport inhibitors inhibit the nitration of PsbO1. Thylakoid membranes were incubated in a buffer bubbled with NO_2_ gas (containing 36.8 µM NO_2_ and 4.35 mM NO_2_^−^). Inhibitors such as 30 µM 3-(3,4-dichlorophenyl)-1,1-dimethylurea (DCMU), 10 mM sodium azide, or 1 mM 1,5-diphenylcarbazide (DPC) were added or not added to the buffer. Proteins were extracted, electrophoresed and Western blotted using a 3-NT-specific antibody followed by quantification of the PSBO1 band intensity. See text for details. Fold-change in PsbO1 band intensity = (intensity of PSBO1 band following incubation in a buffer containing NO_2_ and NO_2_^−^)/(intensity of PSBO1 band before incubation in the buffer). Mean ± SD of three independent experiments. One-way ANOVA with Tukey’s multiple comparison test was used to assess statistical significance: ***, *P* < 0.001.

Thylakoid membranes were incubated in a buffer containing NO_2_ and NO_2_^−^ in the presence or absence of electron transport inhibitors such as 3-(3,4-dichlorophenyl)-1,1-dimethylurea (DCMU), sodium azide and 1,5-diphenylcarbazide (DPC). Proteins were extracted from the treated thylakoid membranes, nitration of PsbO1 was determined by quantification of intensity of PsbO1 band. The results are shown in Figure 6. Fold-change in PsbO1 band intensity is given by (intensity of PSBO1 band after incubation in a buffer containing NO_2_ and NO_2_^−^)/(intensity of PSBO1 band before incubation in the buffer). DCMU inhibits the photosynthetic electron transport by inhibiting binding of plastoquinone [68], and decreased the fold-change in PsbO1 band intensity to about one-fifth of the control value (Figure 6). Azide inhibits the photosynthetic electron transport by inhibiting a variety of reactions, including oxidation of water [69]. Azide also decreased the fold-change in PsbO1 band intensity to one-tenth of the control value (Figure 6). DPC inhibits the photosynthetic electron transport by inhibiting photosynthetic electron flow [70]. DPC decreased the fold-change in PsbO1 band intensity to one-tenth of the control value (Figure 6). Our present findings that nitration of PsbO1 was substantially inhibited by photosynthetic electron transport inhibitors substantiate our postulated nitration mechanism, whereby nitratable tyrosine residue of PsbO1 undergoes one-electron oxidation to tyrosyl radical that is highly reactive with NO_2_ under illumination through PSII photosynthetic electron transport.

**Figure 7:**
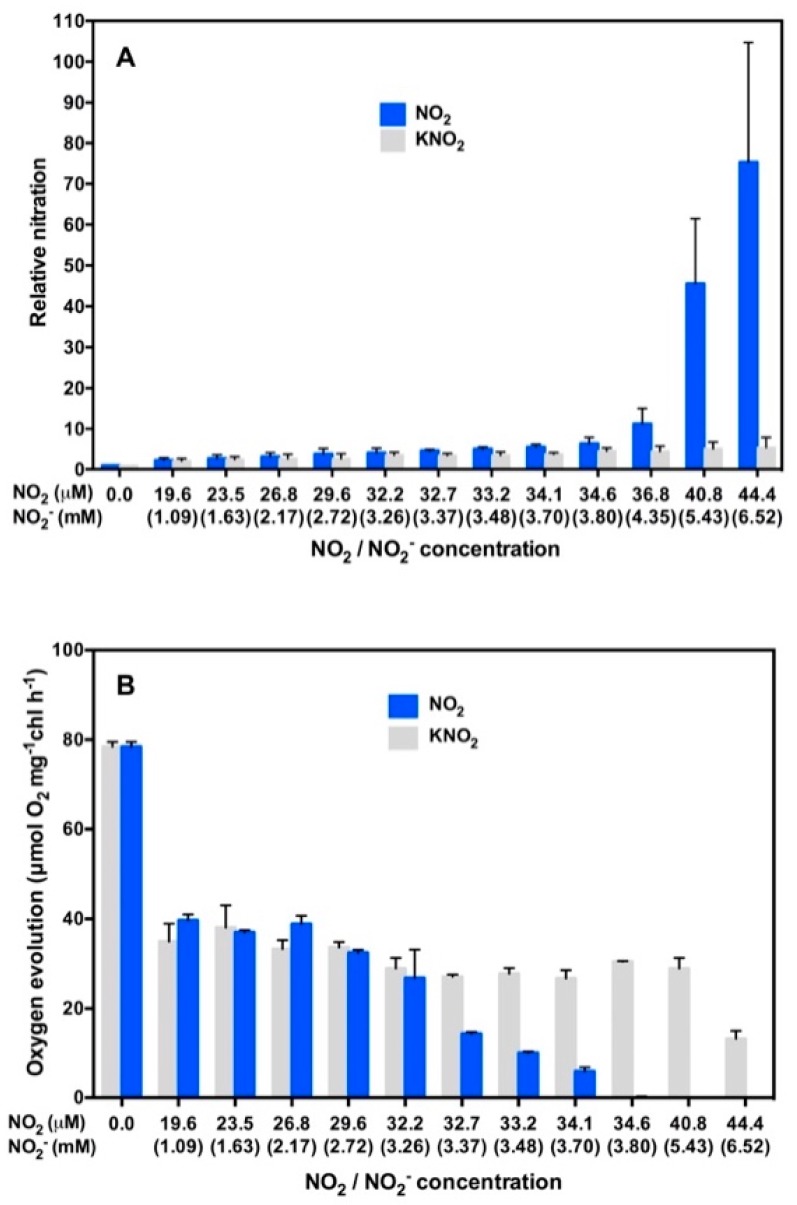
Demonstration that nitration of PsbO1 inhibits oxygen evolution. Arabidopsis thylakoid membranes were incubated in light in a buffer containing NO_2_ and NO_2_^−^ or a buffer containing NO_2_^−^ alone. Incubated thylakoid membranes were divided into equal parts: first one for Western blot analysis and second one for oxygen evolution analysis. (**A**) Relative nitration of PsbO1 as a function of concentrations of NO_2_ or NO_2_^−^. (**B**) Oxygen evolution as a function of concentrations of NO_2_ or NO_2_. The data represent the mean ± SD of three independent experiments.

We next investigated oxygen evolution from isolated thylakoid membranes that had been treated or not treated with a buffer containing NO_2_ and NO_2_^−^ [71]. This buffer contained NO_2_ and NO_2_^−^ as nitrating agent [61]. As it is reported that nitrite anion inhibits PSII to decrease oxygen evolution [72,73,74], it is necessary to separately evaluate these two effects of nitrite on the oxygen evolution. Thylakoid membranes isolated from Arabidopsis leaves were incubated in a buffer containing NO_2_ and NO_2_^−^ or a buffer containing NO_2_^−^ alone in the light or in the dark [71]. After incubation, each of the treated thylakoid membrane samples was divided into two portions. The first portion was analyzed for nitration of PsbO1 by Western blotting using 3-NT-specific antibody. The intensity of the 3-NT-positive PsbO1 band was quantified. Using the second portion, oxygen evolution was quantified [71]. Results are shown in Figure 7.

Incubation of thylakoid membranes in a buffer containing NO_2_^−^ alone at concentrations higher than 3.80 mM NO_2_^−^ did not decrease oxygen evolution to null, but decreased it to one-third to half of the initial value (Figure 7B). On the other hand, oxygen evolution was decreased to almost null when co-existing NO_2_ concentration exceeded 34.6 μM (Figure 7B). This indicates that the effect of NO_2_ higher than 34.6 μM exceeds the effect of NO_2_^−^ to inhibit oxygen evolution when thylakoid membranes were incubated in a buffer containing NO_2_ and NO_2_^−^. This decrease in oxygen evolution is primarily ascribable to nitration of PsbO1 by NO_2_. This substantiates our hypothesis [71,75] that PsbO1 functions as an electron element, like Y_z_ in photosynthetic electron transport.

In light of the present findings regarding the nitration characteristics of ^9^Tyr of PsbO1, selectivity, light dependence, inhibitor-inhibitable and inhibiting oxygen evolution [61,71], and the widely accepted free radical mechanism of tyrosine nitration [45,46], we suggest that illumination induces selective and preferential photo-oxidation of ^9^Tyr of PsbO1, similar to Y_z_. ^9^Tyr may act as an electronic element, similarly to Y_z_ in PSII electron transport chain.

**Figure 8:**
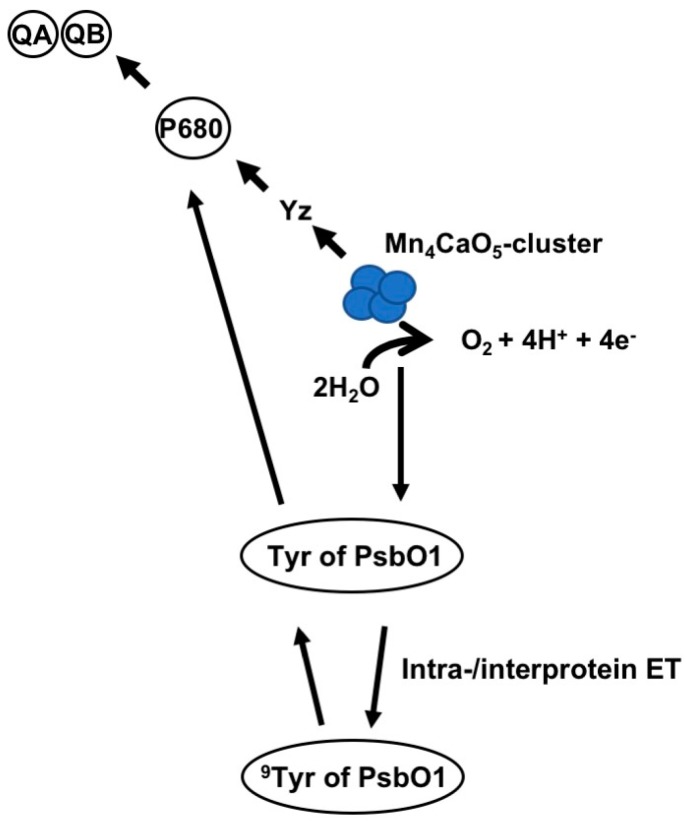
A model to hypothesize a novel role of the ninth tyrosine residue of PsbO1 (^9^Tyr) in photosynthetic electron transport in PSII. Hypothetical long range inter- and intra-molecular electron transfer from manganese cluster to P680+ via ^9^Tyr of PsbO1 supported the ^9^Tyr as a novel electronic element, like Y_z_, in the PSII electron transport.

The 3D structure for plant PSII from pea [76] is the only currently available crystal structure of higher-plant PSII. Using this structure for plant PSII from pea [76] and a molecular graphics software (PyMOL Molecular Graphics System Software, ver. 2.0.7; Schrödinger, New York, NY, USA), ^9^Tyr of PsbO1 and the OEC were calculated to be 36.1Å apart. This is approximately five times greater than the distance between Y_z_ and the OEC Mn_4_ cluster (Mn_4_Ca) (7.5–8.0 Å) [77,78], making it too large for direct interactions [77,79] between the ^9^Tyr of PsbO1 and OEC. However, electron transfer via peptide bonds as distant as more than 40 Å is reported [80]. Furthermore, a 134-Å electron transfer through the helical peptide was also reported [81]. In these cases, the amide groups reportedly act as quantum mechanical hopping sites for electron transfer. Long-range inter-protein electron transfer such as from cytochrome c to cytochrome c peroxidase has also been reported [82]. Moreover, electron transfer between the photosynthetic reaction center and cytochrome c across in *Rhodobacter sphaeroides* has been reported [83]. Taken together, inter-protein electron transfer plays a vital role in cellular metabolism including photosynthesis [82,83]. It is therefore postulated that long-range intra- and inter-protein electron transfer from PSII Mn cluster→^9^Tyr of PsbO1→P680+ (PSII primary electron donor) could support a hypothesis that ^9^Tyr of PsbO1 functions as an electronic element, like Y_z_, in PSII electron transport (Figure 8) [75].

## 5. NO_2_ May Induce Tyrosine Nitration of PYR/PYL/RCAR ABA Receptors Leading to Degradation of the Receptors and Upregulation of TOR, to Stimulate Plant Growth

Our finding that NO_2_-induced nitration of PsbO1 results in reduced oxygen evolution from Arabidopsis thylakoid membranes shows that protein tyrosine nitration alters (downregulates) the physiological function cellular proteins of Arabidopsis leaves. This finding indicates that NO_2_-induced protein tyrosine nitration may be involved in NO_2_-stimulated plant growth. However, as the concentration of NO_2_ used in the study of plant growth (10–50 ppb) was about 800-4000 times lower than that used in the study of protein nitration (40 ppm), further investigations are required to clarify the physiological significance of the NO_2_-mediated nitration of cellular proteins.

**Figure 9:**
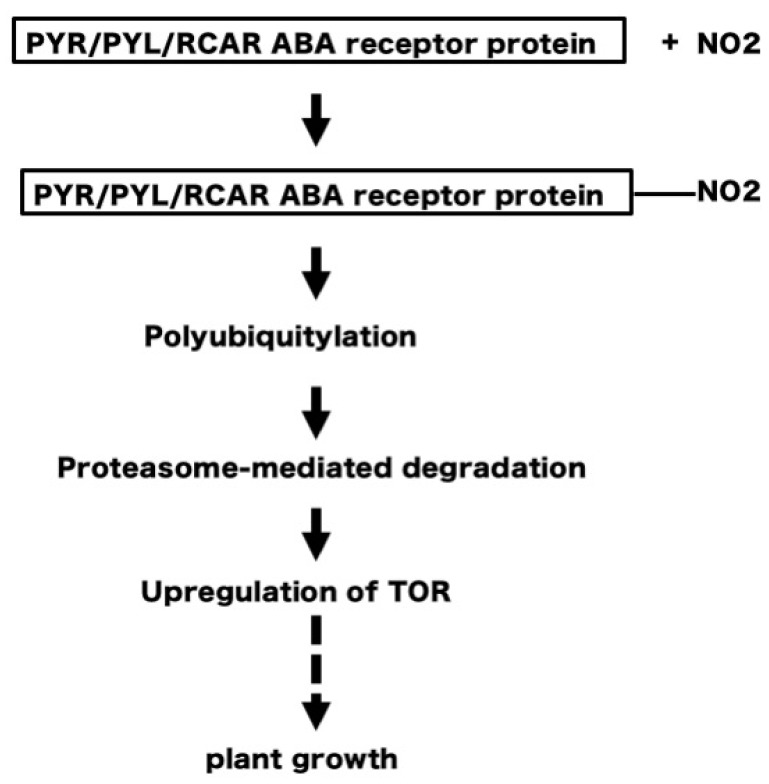
NO_2_ may induce tyrosine nitration of PYR/PYL/RCAR to degrade PYR/PYL/RCAR, and upregulate target of rapamycin (TOR) to stimulate plant growth.

Protein nitration always inhibits protein function in plants [49,84]. In mammalian cells, protein nitration also usually inhibits protein function [45,46,47,50,51], as in plants, but rarely results in gain-of-function of proteins [51]. It remains unknown how protein nitration, a negative regulator, stimulates plant growth. This question is similar to the fundamental and long-standing question as to why an air-pollutant and toxic compounds, such as NO_2_, act as a positive signal for plant growth. Inhibition of negative factors should induce plant growth. Figure 9 depicts a hypothetical model of how NO_2_-induced protein nitration stimulates plant growth. The rationale for this model is as follows:

Plant growth requires the orchestration of a variety of cellular processes, which are controlled by regulatory proteins such as the serine/threonine protein kinase target of rapamycin (TOR), which forms complexes with regulatory proteins [82,85,86]. TOR plays a central role in auxin signal transduction in Arabidopsis [87]. TOR is downregulated by the plant hormone abscisic acid (ABA). ABA detection and signaling are mediated by the pyrabactin resistance1/PYR1-like/regulatory components of the ABA receptor (PYR/PYL/RCAR) family [83,88,89]. Tyrosine nitration of PYR/PYL/RCAR proteins reportedly results in polyubiquitylation and proteasome-mediated degradation [89]. Thus, the degradation of PYR/PYL/RCAR receptor proteins eventually results in upregulation of TOR and stimulation of plant growth [85]. Therefore, it is conceivable that NO_2_ may induce tyrosine nitration of PYR/PYL/RCAR proteins, to degrade these proteins and upregulate TOR regulatory complexes to stimulate plant growth (Figure 9).

## 6. Future Perspectives

In future studies, antibody-assisted proteomic analysis is needed of nitratable proteins in Arabidopsis leaves that are exposed to low concentrations of NO_2_ (10–50 ppb), to ascertain whether PYR/PYL/RCAR proteins from Arabidopsis leaves are nitratable at such low concentrations of NO_2_. Future studies should also investigate whether auxin signal transduction in Arabidopsis leaves [90] is increased following exposure to ambient concentrations of NO_2_. In both cases, special care is needed to ensure that the samples are always isolated from ambient air that contains 10–50 ppb NO_2_. If NO_2_-mediated nitration of PYR/PYL/RCAR proteins cannot be detected, or the involvement of TOR/ABA in NO_2_-mediated plant growth stimulation cannot be ascertained, other target proteins of NO_2_, such as hexokinase-like (HKL) proteins [91], a negative effector of plant growth in Arabidopsis, should be investigated in Arabidopsis leaves.

## Supplementary Materials

The following are available online at https://www.mdpi.com/2223-7747/8/7/198/s1, Table S1: Shoot biomass (mg), total leaf area (mm^2^), and content (µg/shoot) of carbon (C), nitrogen (N), phosphorus (P), potassium (K), calcium (Ca), magnesium (Mg), and sulfur (S) in 5-week-old *Arabidopsis thaliana* C24 plants grown with (+NO_2_-treated plants) and without (–NO_2_ control plants) NO_2_ treatment, Table S2: Correlation analysis between leaf area (RLA) and cell size ( RCS), and that between leaf area (RLA) and cell number RCN), Table S3: Identified nitrated proteins and their electrophoretic and mass spectrometric characteristics in chloroplast proteins extracted from *Arabidopsis thaliana* leaves exposed to NO_2_.
