# Supplementary material for: Nitrogen Dioxide at Ambient Concentrations Induces Nitration and Degradation of PYR/PYL/RCAR Receptors to Stimulate Plant Growth: A Hypothetical Model
Source: Plants (Basel). 2019 Jun 30;8(7):198. doi: 10.3390/plants8070198 (PMC6681506; doi:10.3390/plants8070198)
Supplement: Supplementary file 1 [file plants-08-00198-s001.pdf]

Supplementary materials

Table S1 Shoot biomass (mg), total leaf area (mm^2), and contents (μg/shoot) of seven major elements in 5-week-old *Arabidopsis thaliana* C24 plants grown in the presence (+NO_2-treated plants) or absence (–NO_2 control plants) of NO_2.

	(1) +NO_2- treated plants	(2) –NO_2 control plants	(1) / (2)
Shoot biomass	60.7 ± 8.2***	26.1 ± 3.2	2.3
Total leaf area	(3.43 ± 0.86) × 10^3***	(1.32 ± 0.15) × 10^3	2.6
C	(2.24 ± 0.53) × 10^4*	(9.52 ± 1.52) × 10^3	2.3
N	(4.29 ± 1.15) × 10^3*	1.72 ± 0.26) × 10^3	2.4
P	484 ± 130*	177 ± 28	2.6
K	(1.99 ± 0.48) × 10^3*	903 ± 166	2.1
Ca	(1.29 ± 0.32) × 10^3*	520 ± 77	2.4
Mg	237 ± 49*	121 ± 15	1.9
S	518 ± 106**	202 ± 35	2.5

Shoots of 5-week-old treated and control plants were harvested, washed with pure water and lyophilized. Dry weight of shoots and content of each element were analyzed. Values are means ± SD; $n = 3$. Statistical significance was assessed by Student's t test (*, $P < 0.05$; **, $P < 0.01$; ***, $P < 0.001$).

Table S2. Pearson correlation between leaf area (RLA[1]) and cell size (RCS[2]), and that between leaf area (RLA) and cell number RCN[3]).

		R	*P* value after Bonferroni correction
Log_{10} (RCS)	Leaves (1-25)	0.9	< 0.001
	Leaves (1-11)	0.7	< 0.05
	Leaves (12-25)	0.3	> 0.5
Log_{10} (RCN)	Leaves (1-25)	0.9	< 0.001
	Leaves (1-11)	0.7	< 0.05
	Leaves (12-25)	0.9	< 0.001

[1] RLA = ratio of leaf area of +NO_2-treated plants to that of -NO_2 control plants at corresponding positions.

[2] RCS = ratio of cell size of +NO_2-treated plants to that of -NO_2 control plants at corresponding positions.

[3] RCN = ratio of cell number of +NO_2-treated plants to that of -NO_2 control plants at corresponding positions.

The RLA, RCS and RCN values were transformed into logarithmic values, which were subjected to Pearson's correlation analysis with Bonferroni correction for multiple comparisons.

Table S3. Electrophoretic and proteomic characteristics of chloroplast proteins extracted from *Arabidopsis thaliana* leaves exposed to NO_2.

Spot	Protein	RISI[1)]	RISS[2)]	RISI/RSSI	NCBInr accession #	MOWSE score (s)	Masses matched (%)	Sequence coverage (%)
Chloroplast proteins, stromal and lumenal fraction								
SL7	PSBO1 (OEC 33 kDa subunit)	2.25	1.24	1.8	10177538	85.6	7/14	31.6
SL8	PSBO1 (OEC 33 kDa subunit)	2.21	1.92	1.2	10177538	77.2	11/54	44.6
SL9	PSBO1 (OEC 33 kDa subunit)	13.85	3.78	3.7	10177538	86.7	8/20	36.7
SL10	PSBO1 (OEC 33 kDa subunit)	11.28	8.7	1.3	10177538	44.4	5/24	28.3
SL11	PSBO2 (OEC 33 kDa subunit)	10.25	9.84	1	15230324	1.04e+010	16/50	49.0
SL12	PSBO2 (OEC 33 kDa subunit)	6.58	3.94	1.7	15230324	4.12e+010	17/50	53.0
SL13	PSBP-1 (OEC 23 kDa subunit)	2.84	1.71	1.7	15222166	1.51e+003	6/50	33.0
SL14	PSBP-1 (OEC 23 kDa subunit)	6.26	5.8	1.1	15222166	2.72e+003	7/50	37.0
SL15	PSBP-1 (OEC 23 kDa subunit)	12.46	18.8	0.7	15222166	1.51e+003	5/50	33.0
SL16	PSBP-1 (OEC 23 kDa subunit)	10	17.4	0.6	15222166	6.68e+003	5/50	33.0
SL17	PSBP-1 (OEC 23 kDa subunit)	3.24	5.49	0.6	15222166	7.06e+003	6/50	33.0
SL18	PSBP-1 (OEC 23 kDa subunit)	1.79	8.39	0.2	15222166	1.51e+003	5/50	33.0
SL19	AT4g09650 (F-type H+-transporting ATPase subunit delta)	1.32	0.52	2.5	15233985	4.56e+003	5/50	26.0
SL20	AT4g09650 (F-type H+-transporting ATPase subunit delta)	1.12	0.57	2	15233985	6.61e+004	6/50	36.0
SL21	PRX II E (peroxiredoxin type II E)	3.12	0.47	6.6	15231718	9.12e+004	8/50	45.0
SL22	At4g02530 (thylakoid lumenal 16.5 kDa protein)	3.7	1.4	2.6	15235402	1.08e+006	9/50	55.0
SL23	RCA (RuBisCO activase)	0.34	0.93	0.4	18405145	2.09e+006	11/50	35.0
SL24	PGK1 (phosphoglycerate kinase 1)	0.32	2.69	0.1	15230595	9e+003	7/50	20.0

SL25	CYP38 (peptidyl-prolyl cis-trans isomerase CYP38)	1.29	0.83	1.6	15232123	1.14e+009	11/50	36.0
SL26	At3g55800 (sedoheptulose-1,7-bisphosphatase)	1.06	1.04	1	15228194	85.6	4/12	12.7
SL27	CYP37 (peptidyl-prolyl cis-trans isomerase CYP37)	1.33	1.24	1.1	42564190	2.46e+010	18/50	50.0
SL28	HSP70-6 (Heat shock 70 kDa protein 6)	0.08	0.36	0.2	15233779	1.62e+007	14/50	23.0
SL29	HSP70-6 (heat shock 70 kDa protein 6)	0.33	0.73	0.5	15233779	1.22e+008	16/50	25.0
SL30	CPN60A1 (chaperonin 60 subunit alpha 1)	0.33	0.78	0.4	15226314	1.57e+006	10/50	29.0
SL31	RCA (RuBisCO activase)	1.37	0.36	3.8	30687999	1.71e+003	5/50	16.0
SL32	RCA (RuBisCO activase)	0.94	0.47	2	30687999	9.76e+003	5/50	20.0
SL33	PGK1 (phosphoglycerate kinase 1)	0.34	0.62	0.5	15230595	2.75e+007	11/50	34.0
IS7	PSBO1 (OEC 33 kDa subunit)	23.87	10.32	2.3	15240013	4.68e+004	7/50	28.0
IS8	PSBO1 (OEC 33 kDa subunit)	42.98	15.48	2.8	15240013	1.17e+005	7/50	33.0
IS9	PSBO1 (OEC 33 kDa subunit)	17.52	21.29	0.8	15240013	1.52e+004	7/50	34.0
IS10	PSBO1 (OEC 33 kDa subunit)	5.92	19.35	0.3	15240013	1.11e+006	9/50	32.0
IS19	AT4g09650 (F-type H+-transporting ATPase subunit delta)	1.4	6.45	0.2	15233985	2.27e+005	7/50	40.0
IS20	AT4g09650 (F-type H+-transporting ATPase subunit delta)	2.69	21.29	0.1	15233985	7.98e+005	8/50	40.0
IS34	STR4 (rhodanese-like domain-containing protein 4)	5.62	5.81	1	18411523	3.99e+003	7/50	15.0

[1)]RISI = (intensity of a 3-NT-positive spot on a Western blot)/(sum of intensities of all 3-NT-positive spots on the Western blot) × 100. This estimation was

repeated with three different Western blots with errors of less than 20%.

2)RISS = (intensity of a spot on a SYPRO staining gel)/(sum of intensities of all spots on the SYPRO staining gel) × 100. This estimation was repeated with three separate SYPRO staining gels with errors of less than 20%. The protein spots L9, L14, SL11–25, SL27–33, IS7–10, IS19, 20 and IS34 were analyzed using a Voyager DE STR MALDI-TOF mass spectrometer. The spots L10, L11, L15–17, SL7–10, and SL26 were analyzed using an Ultraflex TOF/TOF mass spectrometer.

3) TM = thylakoid membrane
